# Rising Trend of Substance Abuse Among Older Adults: A Review Focusing on Screening and Management

**DOI:** 10.7759/cureus.76659

**Published:** 2024-12-31

**Authors:** Sima Patra, Sayantan Patra, Reetoja Das, Soumya Suvra Patra

**Affiliations:** 1 Nursing, Brainware University, Kolkata, IND; 2 Interventional Radiology, Rabindranath Tagore International Institute of Cardiac Sciences, Kolkata, IND; 3 Ophthalmology, Regional Institute of Ophthalmology, Medical College and Hospital, Kolkata, IND; 4 Psychiatry and Behavioral Sciences, Nassau University Medical Center, East Meadow, USA

**Keywords:** alcohol abuse, assist, audit, cage questionnaire, illicit drugs, mast-g, older adults, prescription medication abuse, substance abuse, tobacco use

## Abstract

There is undoubtedly an alarmingly rising trend of substance use among older adults. This has necessitated a paradigm shift in healthcare and propelled strategies aimed at effective prevention and screening. Age-related physiological changes, such as diminished metabolism and increased substance sensitivity, make older adults particularly vulnerable to adverse effects of substances. This not only has adverse psychological consequences but also physical consequences like complicating chronic illnesses and harmful interactions with medications, which lead to increased hospitalization.

Standard screening tools can identify substance use disorders (SUDs) in older adults. Tools like the Cut-down, Annoyed, Guilty, and Eye-opener (CAGE) questionnaire and Michigan Alcohol Screening Test-Geriatric (MAST-G) are tailored to detect alcoholism, while the Alcohol, Smoking, and Substance Involvement Screening Test (ASSIST) and Alcohol Use Disorders Identification Test (AUDIT) assess abuse of illicit and prescription drugs. Since older adults are more socially integrated, screening should be done using non-stigmatizing and non-judgmental language.

Prevention strategies include educational programs, safe prescribing practices, and prescription drug monitoring. Detection of substance abuse should be followed by brief interventions and specialized referrals. In conclusion, heightened awareness, improved screening, and preventive measures can mitigate substance abuse risks in this demographic. Prioritizing future research on non-addictive pain medications and the long-term effects of substances like marijuana seems justified.

## Introduction and background

The world is witnessing an increasing trend towards substance use among older adults, especially the baby boomer generation. The concept of "unhealthy substance use" encompasses risky behaviors, including excessive alcohol consumption, tobacco use, illegal drugs, and the misuse of prescription medications. Historically, older adults tended to reduce substance use over time, but this scenario is shifting due to changing attitudes and social norms [1,2].

The Diagnostic and Statistical Manual of Mental Disorders, Fifth Edition (DSM-5) now defines substance use disorders (SUDs) as a pattern of substance use that leads to significant impairment. Changes in the DSM-5 terminology have streamlined the identification and classification of these disorders by eliminating terms like "substance abuse" and "dependence" [3]. The risk factors for substance abuse in older adults include chronic pain syndromes, general debilitation, polypharmacy, physical disability, other co-morbidities, prior substance abuse, and social isolation. Demographics that are commonly associated with SUDs in old age include male sex, Caucasian ethnicity, unmarried or divorced status, recent bereavement, avoidant coping techniques, and even being not religiously active [4].

Diagnosing substance abuse in older adults can be very challenging. The condition is often underdiagnosed or frequently misdiagnosed as dementia, anxiety, and/or depression. The impact of substance use in older adults is especially critical because they have higher rates of chronic diseases and often take multiple medications, creating risks of adverse drug interactions and exacerbated health conditions. The most common of these conditions include fractures from increased falls and worsening of incontinence, nutrition, memory and sleep problems, anxiety, and depression. This context makes it essential to implement screening and preventative strategies to mitigate the potential public health impact [4-6].

**Figure 1:**
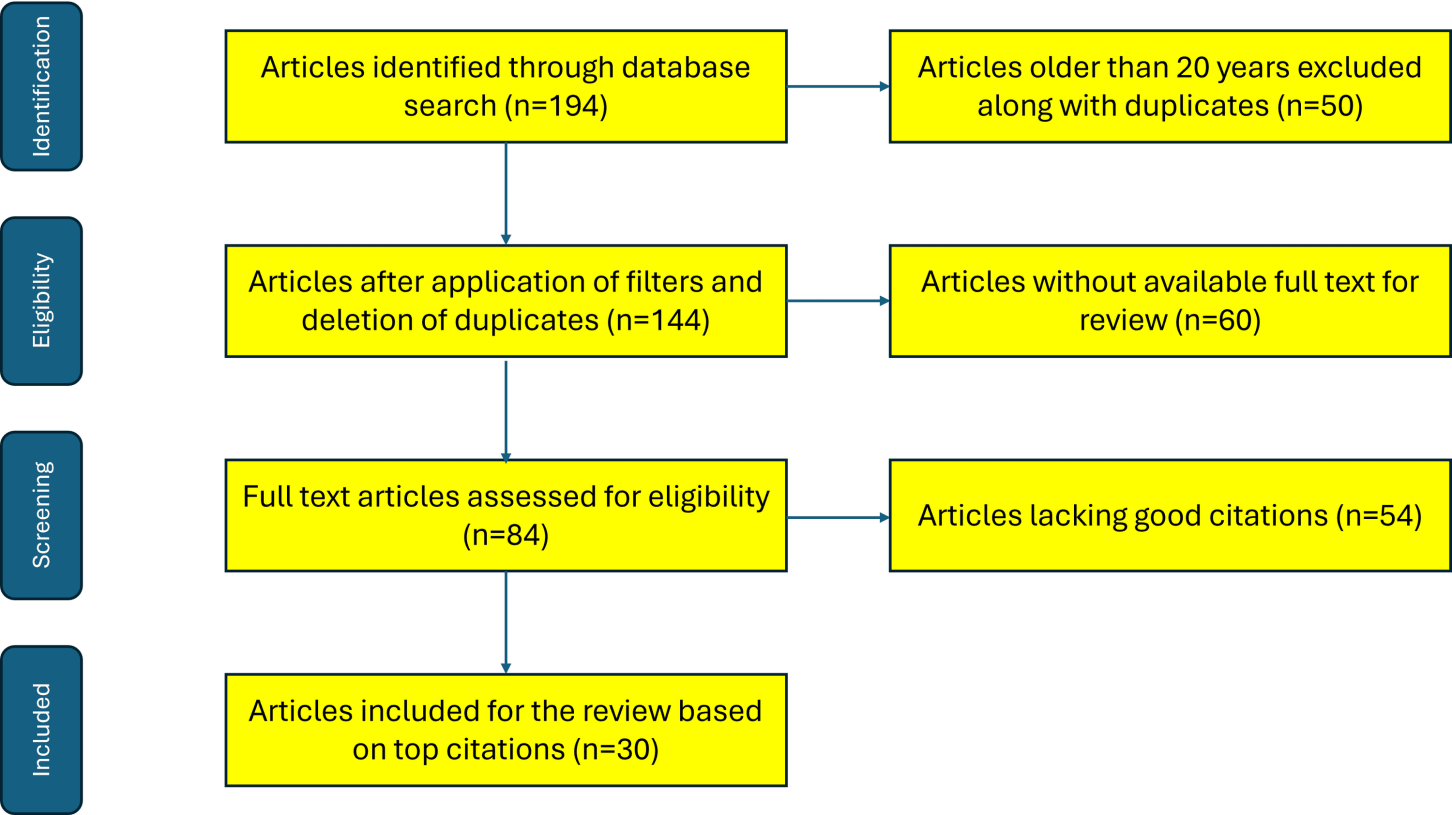
PRISMA flow diagram showing the methodology of literature review for the present article PRISMA: Preferred Reporting Items for Systematic Reviews and Meta-Analyses The figure was created by the authors.

Prior literature from the past two decades that pertains to issues related to substance abuse in the elderly was included in this review. Literature from the internet (including PubMed and Scopus databases), particularly for the latest versions of questionnaires/tools for screening substance abuse, was also included in this review. The literature search was performed using keywords: "older adults", "substance abuse", "alcohol abuse", "tobacco use", "illicit drugs", "prescription medication abuse" and names of all the screening tools/questionnaires pertaining to these substances. Literature older than 20 years and older versions of the questionnaires were excluded from the present review. A Preferred Reporting Items for Systematic Reviews and Meta-Analyses (PRISMA) flow diagram depicting the methodology of the literature search is shown in Figure 1.

The article was presented as an e-poster, in brief, at the 3^rd^ International Virtual Conference on "Partnership and Collaborative Work for Nursing Practitioners: In Managing Behavioral Addiction and Substance Use Disorders," held on November 7, 2024.

## Review

Major substances of abuse - a detailed foray into the prevalence of use and adverse effects

Recent large cohort researches indicate that substance use among older adults has been increasing, with notable changes in the types of substances used. Data from the Treatment Episode Data Set - Admissions (TEDS-A) reveals that from 2008 to 2018, the percentage of admissions for adults aged 55 and over rose dramatically from 9.04% to 15.64%. Notably, admissions for illicit drug use surpassed those for alcohol by 2018, highlighting a shift in substance use patterns within this demographic [2,7].

The statistical evidence is alarming. The substances in order of frequency among patients admitted as a direct consequence of substance abuse are alcohol, cocaine, cannabis, opioids, and benzodiazepines. Alcohol remains the most commonly reported substance (66.7% of admissions). Cocaine, reported by 14.8% of older adults, is the second most common substance abused in the United States (US). Cannabis is the third most common, reported by 14.1%. Opioid misuse was observed in 8.6% of cases and benzodiazepine abuse is reported by 2.4% [7]. This increase in in-patient healthcare reflects broader trends in substance use, with older adults exhibiting higher rates of earlier-life substance use that may continue into later years [2].

Alcohol

Unhealthy alcohol use is widely common globally and is one of the leading preventable causes of death. Alcohol continues to be the substance most commonly used by older adults, and its use is expected to rise even more. The National Survey on Drug Use and Health (NSDUH) in 2013/2014 discovered that 62.1% of the elderly consume alcohol. Moreover, binge drinking is reported by 21.5% of elderly men and 9.1% of elderly women, while alcohol use disorders impact 5.1% of men and 2.4% of women in this age group. The data shows a significant increase in binge drinking (up by 19.2%) and alcohol use disorders (up by 23.3%) among older adults compared to the previous decade [2,8]. Consuming a moderate amount of alcohol, which is defined as one drink per day, has demonstrated possible health advantages for certain elderly individuals, including lower rates of illness and death. Nevertheless, as alcohol consumption becomes more intense, risks also rise substantially. As individuals age, their decreased liver function, lower body water content, and heightened neuronal sensitivity result in heightened alcohol sensitivity and reduced tolerance. Due to this, the elderly are at a higher risk of experiencing negative consequences from alcohol, potentially exacerbating issues such as high blood pressure, irregular heartbeats, bleeding in the brain, liver damage, stomach bleeding, and specific types of cancer. Alcohol can also dangerously interact with medications often prescribed to elderly individuals, increasing the chance of negative results [9-11]. Because of these reasons, the National Institute on Alcohol Abuse and Alcoholism (NIAAA) suggests that older adults should adhere to lower alcohol consumption limits, including no more than three drinks in one day and no more than seven drinks per week for healthy individuals over the age of 65 who are not using medications. Yet, a number of elderly individuals and their medical professionals might not be informed about these recommendations, causing them to consume alcohol at levels that could be harmful without being aware of the dangers involved [2,12].

Tobacco

Around 8.4% of individuals who are 65 years old and above are currently smoking. Unlike alcohol, smoking has no positive effects on health and is closely linked to various health dangers, especially to heart and lung health, along with an increased likelihood of cancer and death. In older individuals, smoking is associated with a decrease in cognitive function, restrictions in daily activities, and heightened susceptibility to age-related illnesses. Even though many older smokers have attempted to quit several times without success, the advantages of quitting are still significant regardless of age. Studies indicate that stopping smoking, even for individuals over 65 years old, results in much lower death rates than those who persist in smoking, highlighting the importance of efforts to help older individuals quit smoking [13-16].

Illegal Drugs

The effects of alcohol as well as other substances like benzodiazepines, opioids, and marijuana, are magnified by physiological changes in aging. Due to changing attitudes, legalization for recreational use, and growing medical use, marijuana has become more common among older adults. The 2012/2013 NSDUH discovered that 4.8% of elderly individuals admitted to using marijuana within the last year, showing a significant surge from prior periods, with a 57.8% boost in consumption for individuals aged 50-64 and a 250% surge for adults over 65 [6,17]. Research on the safety and effectiveness of marijuana and cannabis products for medical conditions like seizures, multiple sclerosis, and chronic pain is limited, particularly in older adults, despite potential benefits. Certain research indicates that marijuana usage can increase heart rates, potentially heightening the possibility of cardiovascular disorders, while smoking it may elevate the chances of lung disease and respiratory infections. Furthermore, the use of marijuana has been linked to a higher chance of cerebrovascular events, interactions with other drugs, and negative impact on cognitive abilities. Additional research is necessary to assist healthcare professionals in better evaluating the risks and benefits of marijuana for older adults, particularly those with various chronic illnesses and heavy use of prescription medications [18,19]. Few older adults use other illegal drugs, as only 0.41% of those aged 50 and above reported using cocaine in the 2005/2006 NSDUH. Yet, research conducted in specific urban hospitals has revealed an increased occurrence of older adults using cocaine, with rates ranging from 2% to 2.3%. Cocaine presents unique dangers to the cardiovascular and cerebrovascular systems of elderly people, which may result in disability or sudden death. While heroin usage is uncommon among older adults in the general population (0.03% past-year use), certain regions, such as New York City, have seen significant rises in its use [6].

Prescription Drugs

Elderly individuals are significant users of drugs, with a minimum of 87% of individuals between the ages of 62 and 85 taking at least one prescribed medication and 36% taking five or more. This frequent use raises their chances of experiencing drug interactions and possible abuse. Research has shown that approximately 25% of elderly individuals are taking psychoactive drugs that can be misused, leading to a substantial rise in emergency room visits due to prescription misuse among those aged 50 and above - an increase of 121% between 2004 and 2008. Prescriptions for anxiety or insomnia medications are frequently misused, followed closely by pain relievers [6,20]. The presence of prescription opioids has greatly increased, quadrupling over the last 10 years and exerting a significant impact on the elderly population. Around 1.4% of individuals aged 50 and above acknowledge using prescription opioids for non-medical purposes, with older adults being at a heightened risk of opioid overdose and higher mortality rates linked to opioid use disorders. Further investigation is required to determine the effect of chronic opioid use on the health of elderly individuals, including its influence on chronic diseases, functional capacities, and healthcare usage [21,22]. Benzodiazepines are often prescribed to older adults and are commonly misused due to their high potential for misuse. Even though there are known dangers like falls, cognitive issues, delirium, and negative reactions with other medications, the use of benzodiazepines continues to be prevalent, with prescription rates as high as 30% in certain groups. Long-acting benzodiazepines are not recommended as the initial treatment for conditions such as agitation or insomnia in elderly individuals, and older adults should avoid benzodiazepines as their primary therapy due to increased risks. Healthcare professionals should be careful when prescribing benzodiazepines to elderly patients to avoid negative results [6,23,24].

Screening tools for SUDs in the elderly

Sympathy and empathy towards the patient’s condition are imperative in the initiation of psychiatric management of SUDs. Often, the patient’s medical condition, sleep quality, pain levels, physical capabilities (or lack thereof), and use of over-the-counter medication have to be taken into consideration before clinical suspicion of substance abuse. Only after optimizing these underlying physical conditions can one confidently suspect substance abuse and offer diagnostic questionnaires or treatment for the same [2,18].

**Figure 2:**
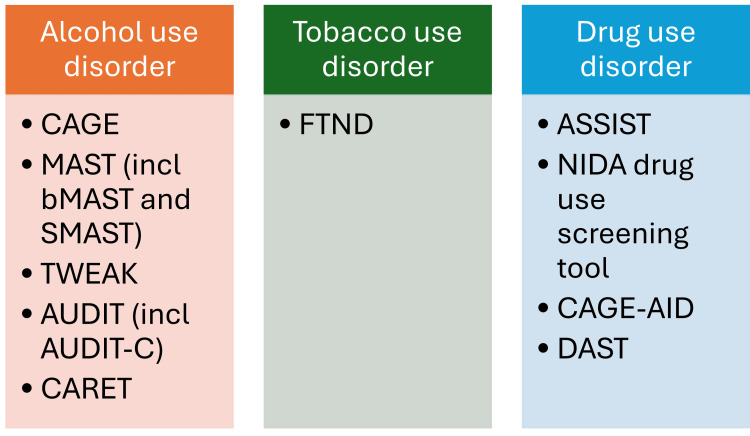
Tools (questionnaires) commonly used for screening substance abuse in the elderly CAGE: Cut-down, Annoyed, Guilty, and Eye-opener; MAST: Michigan Alcohol Screening Test; bMAST: Brief MAST; SMAST: Short MAST; MAST-G: MAST-Geriatric; TWEAK: Tolerance, Worried, Eye-opener, Amnesia, and K/Cut-down; AUDIT: Alcohol Use Disorders Identification Test; AUDIT-C: AUDIT-Consumption; CARET: Comorbidity Alcohol Risk Evaluation Tool; FTND: Fagerstrom Test for Nicotine Dependence; ASSIST: Alcohol, Smoking, and Substance Involvement Screening Test; NIDA: National Institute on Drug Abuse; CAGE-AID: CAGE-Adapted to Include Drugs; DAST: Drug Abuse Screening Test (DAST) The figure was created by the authors.

A vast array of tools are available for identification of SUDs (Figure 2). Most of the tools are independent of the age of the subject; however, some modified questionnaires specific to the elderly population do exist. Although not exhaustive, the most commonly used tools for screening can be broadly classified according to the substance they are used to screen. Alcohol use disorder tools include Cut-down, Annoyed, Guilty, and Eye-opener (CAGE) questionnaire, Michigan Alcohol Screening Test (MAST) (including brief MAST (bMAST), short MAST (SMAST), MAST-Geriatric (MAST-G), and Veterans Alcoholism Screening Test (VAST)), Tolerance, Worried, Eye-opener, Amnesia, and K/Cut-down (TWEAK) questionnaire, Alcohol Use Disorders Identification Test (AUDIT) (including AUDIT-Consumption (AUDIT-C)), and Comorbidity Alcohol Risk Evaluation Tool (CARET). Among tobacco use disorder tools, the Fagerstrom Test for Nicotine Dependence (FTND) is the most commonly employed. For drug use disorder, a number of tools are available, notable of which include Alcohol, Smoking, and Substance Involvement Screening Test (ASSIST), National Institute on Drug Abuse (NIDA) Drug Use Screening Tool, CAGE-Adapted to Include Drugs (CAGE-AID) questionnaire, and Drug Abuse Screening Test (DAST).

Alcohol use disorder tools

CAGE Questionnaire

The CAGE questionnaire is the most common screening tool for alcohol misuse and dependency, applicable to any age group. Developed in 1968 at North Carolina Memorial Hospital, the CAGE questionnaire consists of four simple questions that help identify individuals who may have problems with alcohol. The acronym "CAGE" stands for cut down, annoyed, guilty, and eye-opener (Table 1).

**Table 1:** The CAGE questionnaire for alcohol use disorder CAGE: Cut-down, Annoyed, Guilty, and Eye-opener

Acronym letter	Summarized epithet	Question format
C	Cut-down	Have you ever felt you should cut down on your drinking?
A	Annoyed	Have people annoyed you by criticizing your drinking?
G	Guilty	Have you ever felt guilty about drinking?
E	Eye-opener	Have you ever had a drink first thing in the morning to steady your nerves or get rid of a hangover?

Each "yes" response scores one point, and a score of two or more may indicate a potential problem with alcohol. This tool is often used in clinical settings where quick assessment is necessary. The CAGE questionnaire has excellent discriminative validity and clinical utility. However, being a self-reported questionnaire with an obvious end point of questions, responses are subject to social desirability bias (as people are uncomfortable or stigmatized). Often anonymous or online responses are relatively free of these biases [2,25].

MAST

MAST was initially developed by Selzer in 1971. It is one of the top 100 screening tests for assessment of alcoholism and alcohol-related disorders. In addition, there are seven brief and alternative versions of MAST [26]. MAST was developed as a quick screening test using paper and pencil format. The questionnaire consists of 25 "yes/no" questions that inquire about a person's past and current alcohol use and its adverse effects. There are two different methods of scoring, namely the unit scoring procedure, in which one is noted for each response, or the weighted scoring system, in which two or five points are assigned for only highly discriminative items [2,6,26].

The scoring takes approximately five minutes and is hence very useful in primary care settings. A cut-off score of five is recommended for the unit scoring method for the identification of problem drinking. A score of three to four indicates possible problem drinking. In the weighted scoring method, a score of seven for alcoholism and a score of four to six for possible problem drinking is considered standard. Both the scoring systems are equally weighted at r=0.99 from most studies and are, therefore, considered equally effective [6]. There are a few drawbacks of MAST, namely indistinct phasing of items, focus on lifetime occurrence of events, and dichotomous response format, which makes it difficult to differentiate between current and past drinking habits [26].

Alternative and short versions of MAST

MAST has several alternative and/or shorter versions designed to efficiently assess alcohol-related problems. Although the original MAST consists of 25 questions, these shorter adaptations aim to maintain the instrument's effectiveness while reducing the time required for administration. Notable alternative and/or shorter versions of MAST include MAST-Alcohol/Drug (MAST-AD), MAST-10, MAST-13, and MAST-5. MAST-G and SMAST-Geriatric (SMAST-G) are versions developed for the geriatric population specifically. [6, 27]

MAST-AD version modifies the original MAST by changing references from "alcohol" to "alcohol and/or drugs." It consists of 24 items and is particularly useful for assessing substance use beyond just alcohol.

MAST-10 (also called bMAST) is a 10-item version that retains key questions from the original MAST, focusing on critical indicators of alcohol misuse. This version is designed for quick screening in clinical settings where time is limited. Similarly, MAST-13 (also called SMAST) is a relatively longer abbreviated version that includes 13 items, selected based on their psychometric properties and relevance in identifying problematic drinking behaviors. The questions in SMAST and bMAST are listed in Table 2.

**Table 2:** Questions in SMAST (13 items) and bMAST (10 items) SMAST: Short Michigan Alcohol Screening Test; bMAST: Brief Michigan Alcohol Screening Test

Questions in SMAST	Questions in bMAST
Do you feel you are a normal drinker?	Do you feel you are a normal drinker?
Does your spouse (or a close friend) ever worry or complain about your drinking?	Does your spouse (or a close friend) ever worry or complain about your drinking?
Have you ever felt guilty about your drinking?	Have you ever felt guilty about your drinking?
Have you ever awakened or gone to work in the morning with a hangover?	Have you ever awakened or gone to work in the morning with a hangover?
Do you drink to relax?	Do you drink to relax?
Have you ever had a blackout?	Have you ever had a blackout?
Have you ever neglected your family or lost time from work due to drinking?	Have you ever neglected your family or lost time from work due to drinking?
Have you ever gotten into trouble because of drinking?	Have you ever gotten into trouble because of drinking?
Has your drinking caused trouble with your spouse (or a close friend)?	Has your drinking caused trouble with your spouse (or a close friend)?
Have you ever been arrested for drunken driving?	Have you ever been arrested for drunken driving?
Have you ever gone to any doctor or clinic about your drinking?	
Do you ever drink alone?	
Have you ever thought about cutting down on your drinking?	

For both bMAST and SMAST, a score of zero to two indicates no alcohol problem, three indicates a possible alcohol problem, and four or more indicates a definite problem [27].

MAST-5 is an ultra-short version of MAST containing only five items. It provides a rapid assessment tool that can be used in various settings, including primary care and emergency departments.

These alternate and short versions are particularly beneficial in situations where a full assessment may not be feasible, such as in busy clinical environments or initial screenings. They maintain a focus on identifying significant alcohol-related issues while allowing for quicker evaluations. The most commonly used MAST modifications are summarized in Table 3.

**Table 3:** Summary of alternate/short versions of MAST MAST: Michigan Alcohol Screening Test; bMAST: Brief MAST; SMAST: Short MAST; MAST-AD: MAST-Alcohol/Drug

Alternate/short version	Number of items/questions	Modification and rationale
MAST-AD	24	Assessment of both alcohol and substances beyond alcohol
MAST-10 or bMAST	10	Focuses on critical indicators of alcohol use disorder. Designed for quick screening
MAST-13 or SMAST	13	Focuses on critical indicators of alcohol use disorder. Designed for quick screening
MAST-5	5	Very rapid assessment for emergency departments and primary care settings

Research indicates that these shorter versions retain adequate reliability and validity compared to the full-length MAST. Short versions of MAST provide flexible options for screening alcohol use disorders while ensuring the effective identification of individuals who may benefit from further assessment or intervention. However, practitioners should consider the context of use and the potential trade-offs when choosing which version to administer [2,6,7,27]. 

MAST-G and SMAST-G

MAST-G and SMAST-G are modified versions of the original MAST, specifically designed to assess alcohol use disorders in older adults. This adaptation acknowledges the unique drinking patterns and health considerations prevalent in the geriatric population [28]. The key alterations of MAST-G and SMAST-G from their initially developed counterparts are summarized in Table 4.

**Table 4:** Key alterations in MAST-G and SMAST-G compared to MAST and SMAST, respectively MAST: Michigan Alcohol Screening Test; SMAST: Short MAST; MAST-G: MAST-Geriatric; SMAST-G: SMAST-Geriatric

Key alterations	Description
Target population	Tailored for older adults, taking into account their specific social and health contexts
Shortened format	SMAST-G has 10 items, making it more concise and suitable for quick assessments. MAST-G has 24 items like the original MAST
Focus on relevant issues	Designed to address common concerns related to alcohol use in older adults, such as medication interactions, cognitive decline, and social isolation
Scoring system	In MAST-G, like the original MAST, higher scores indicate greater potential for alcohol-related problems
Psychometric properties	MAST-G is validated for use in geriatric populations, with reliability and validity comparable to the original MAST

MAST-G is particularly useful in various healthcare settings, including primary care, where geriatric mental health services are the focus. Both MAST-G and SMAST-G provide a much more focused approach to screening for alcohol use disorders among only older adults, keeping nuances specific to older adults in mind [27,28].

VAST

VAST is a modification of MAST, specifically designed to identify alcohol use disorders among veterans. The key distinguishing advantage of VAST is that it allows a distinction between past and present alcoholism. The responses are categorized into use up to one year ago, between one to five years ago, and over five years ago. VAST overcomes the general shortcomings of MAST and is considered a refined but time-consuming version, especially for the elderly [29].

TWEAK Questionnaire

The TWEAK questionnaire is a brief, five-item questionnaire designed to screen for alcohol use disorders. "TWEAK" is an acronym for the following questions: tolerance, worried, eye-opener, amnesia, and ​​​​k/cut down.The detailed version is presented in Table 5. 

**Table 5:** Detailed description of the TWEAK questionnaire TWEAK: Tolerance, Worried, Eye-opener, Amnesia, and K/Cut-down

Acronym letter	Description	Question
T	Tolerance	How many drinks does it take to feel high?
W	Worried	Have close friends or relatives worried or complained about your drinking in the past year?
E	Eye-opener	Do you sometimes take a drink first thing in the morning when you wake up?
A	Amnesia	Has a friend or family member ever told you about things you said or did while you were drinking that you could not remember?
K	K/Cut-down	Do you sometimes feel the need to cut down on your drinking?

The TWEAK questionnaire is scored on a seven-point scale. Tolerance and worried are assigned two points for a positive response. Eye-opener, amnesia, and k/cut-down are assigned one point for a "yes" response to each. A score of two or more indicates a potential alcohol use disorder and may warrant further assessment or intervention [30,31].

Although the TWEAK questionnaire was initially intended for the identification of hazardous drinking behaviors in women who are pregnant or may become pregnant for primordial prevention of fetal alcohol spectrum disorders (FASD), it can be applied to the geriatric population as well. The TWEAK questionnaire has been shown to have good reliability and validity in detecting alcohol use disorders among various populations, in addition to prenatal care settings. Widespread use of the TWEAK questionnaire as a screening tool may be encouraged outside of prenatal settings, in primary care, emergency departments, and specialty clinics [32].

AUDIT

AUDIT is a widely recognized screening tool developed by the World Health Organization (WHO) to identify individuals with alcohol use disorders and hazardous drinking behaviors. It is designed for use in various healthcare settings and can be administered to individuals regardless of their current drinking status [33].

The AUDIT tool comprises 10 questions that assess alcohol consumption, drinking behavior, and alcohol-related problems over the past year. The 10 questions in AUDIT are detailed in Table 6. These questions cover key aspects of alcohol abuse like frequency of alcohol consumption, quantity of alcohol consumed on a typical drinking day, frequency of heavy drinking episodes, and alcohol-related consequences, such as guilt or regret after drinking [33].

**Table 6:** Detailed description of the 10-item AUDIT AUDIT: Alcohol Use Disorders Identification Test

Question number	Question
1	How often do you have a drink containing alcohol?
2	How many standard drinks containing alcohol do you have on a typical day when you are drinking?
3	How often do you have six or more drinks on one occasion?
4	How often have you failed to do what was normally expected of you because of drinking?
5	How often have you neglected your family or friends because of drinking?
6	How often have you felt guilty or remorseful after drinking?
7	How often have you not been able to stop drinking once you have started?
8	How often have you not been able to do what you planned to do because of drinking?
9	Have you or someone else been injured as a result of your drinking?
10	Has a relative or friend or a doctor or another health worker been concerned about your drinking or suggested you cut down?

Each question is scored from zero to four, leading to a total score ranging from zero to 40. Higher scores indicate a greater likelihood of hazardous or harmful drinking. Scores of zero to seven indicate low risk, scores of eight to 15 indicate increasing risk, scores of 16 to 19 indicate high risk, and scores of 20 and above indicate possible dependence. A score of eight or above indicates hazardous alcohol use [2,6].

The primary aim is to detect early signs of alcohol misuse and provide a basis for further assessment and intervention. Like all previous short questionnaires, AUDIT is aimed at use in primary care settings, emergency departments, and mental health services where detailed history-taking is not practical [6].

The most important advantage of AUDIT is that it can either be self-administered or conducted as an interview by a healthcare professional, making it flexible and adaptive for various clinical scenarios. Inevitably, AUDIT remains an essential screening tool for healthcare providers, regardless of the age of the patient. AUDIT's structured format, robust scoring system, and proven psychometric properties across multiple studies make it an effective instrument for identifying individuals likely to benefit from further evaluation or intervention [6].

Modifications of AUDIT

Numerous AUDIT modifications have been proposed to better suit specific populations and clinical settings. Three considerations are usually taken into account for modifications of AUDIT in different populations or sub-groups of populations: cultural sensitivity, language barriers, and contextual factors. 

Cultural factors that may influence drinking behaviors and attitudes toward alcohol may need modifications of AUDIT. In addition, AUDIT should be administered in the patient's preferred language to ensure accurate self-reporting. When interpreting AUDIT scores, it is important to consider the individual's social, cultural, and environmental context.

Common adaptations of the 10-item AUDIT include the AUDIT-C and language modifications for specific populations. While modifications can enhance the effectiveness of AUDIT, it remains essential to use the tool appropriately and interpret results alongside other clinical information. As in all cases, consultation with a mental healthcare professional is required for a tailored treatment plan.

AUDIT-C is a condensed version of AUDIT, consisting of just three questions. AUDIT-C primarily focuses on the frequency of heavy drinking and is particularly effective for quick screenings in primary care environments. A score of five or higher indicates risky alcohol behavior.

Other modifications for specific populations include those for older adults, women, and culturally/ethnically different groups. AUDIT may need adjustments to reflect age-related changes in drinking habits and health issues. Adjustments of questions to reflect the adverse effects on geriatric diseases, existing conditions, or drug interactions are important in this age group. Similarly, modifications specific to women might be necessary to take into consideration differences in drinking patterns and alcohol metabolism between genders. Adjustments in wording to ensure the cultural relevance of questions may be required to ensure accurate assessments across diverse populations [2,34,35].

CARET

CARET is a screening tool designed to identify at-risk drinking in middle-aged and older adults (aged 50 years or older). This unique tool goes beyond assessing simple alcohol consumption by considering a broader range of factors that can contribute to alcohol-related harm. It considers factors like alcohol consumption and its adverse effects on medical comorbidities and/or psychiatric manifestations. CARET also considers the use of medications that can interact with alcohol, potentially leading to adverse effects [34,35].

The essential components of CARET assessment are alcohol consumption assessment, medical co-morbidity evaluation, and medication review. Alcohol consumption assessment includes quantity and frequency of consumption, along with binge drinking episodes. Medical co-morbidity evaluation includes the presence of medical conditions like hypertension, diabetes, heart disease, and psychiatric disorders such as depression and anxiety. Medication review includes assessment for medications that can interact with alcohol, such as sedatives, blood thinners, anti-hypertensives, anti-depressants, and certain pain relievers.

Individuals are classified as at-risk if they meet three essential criteria: exceeding specific drinking limits, combining alcohol with certain medical conditions or medications, and experiencing alcohol-related symptoms like sleep disturbances, memory problems, or digestive issues. By considering these multiple factors, CARET can identify individuals who may not be flagged as at-risk based solely on their drinking patterns. This comprehensive approach allows for earlier intervention and tailored treatment plans to address alcohol-related problems and reduce associated health risks.

Compared to all other scoring systems like CAGE or AUDIT-C, CARET supplements the normal tally of questions with additional questions regarding individual health conditions, medication use, and specific alcohol-related risk behaviors like drunk driving. This enables healthcare professionals to significantly improve the accuracy of screening and identify a broader range of older adults at risk. This proactive approach can lead to earlier interventions and ultimately reduce the negative consequences of alcohol misuse among older populations [35].

Tobacco use disorder tools

FTND

FTND is a widely used tool to assess the level of nicotine addiction. It consists of six questions that measure different aspects of smoking behavior, as presented in Table 7 [36]. 

**Table 7:** The six-item FTND in detail FTND: Fagerstrom Test for Nicotine Dependence

Question number	Essence of question	Question body
1	Time to the first cigarette	How soon after waking up do you smoke your first cigarette?
2	Difficulty refraining	Do you find it difficult to refrain from smoking in places where it is forbidden?
3	Most important cigarette	Which cigarette would you hate most to give up?
4	Number of cigarettes per day	How many cigarettes do you smoke per day?
5	Morning smoking	Do you smoke more frequently during the first hours after waking up than during the rest of the day?
6	Smoking when ill	Do you smoke if you are so ill that you are in bed most of the day?

Each question is scored on a scale of zero to three, with higher scores indicating a higher level of nicotine dependence. The total score ranges up to 18, and a higher score suggests a stronger addiction to nicotine. FTND is highly reliable and finds use in assessing the severity of nicotine addiction, monitoring treatment progress, and predicting quitting success [36,37].

Drug use disorder tools

ASSIST

ASSIST is a screening tool designed to identify individuals at risk of substance use disorders. It assesses a wide range of substances, including alcohol, tobacco, cannabis, cocaine, amphetamines, inhalants, sedatives, hallucinogens, and opioids [38].

ASSIST is an easy-to-administer tool, which can be completed in a relatively short time, and has a number of important stand-out features. It is a versatile tool covering a broad spectrum of substances. Additionally, it provides a risk score for each substance, allowing for tailored interventions based on the magnitude of risk. Based on the risk score, ASSIST offers recommendations for appropriate interventions, such as brief interventions or referral to specialist treatment. The eight-item ASSIST is detailed in Table 8.

**Table 8:** Detailed description of the eight-item ASSIST ASSIST: Alcohol, Smoking, and Substance Involvement Screening Test

Question number	Question	Response type for each substance
1	In your life, which of the following substances have you ever used?	“Yes” or “No”
2	In the past three months, how often have you used the substances you mentioned?	“Never”, “once or twice”, “monthly”, “weekly” or “daily or almost daily”
3	During the past three months, how often have you had a strong desire or urge to use?	“Never”, “once or twice”, “monthly”, “weekly” or “daily or almost daily”
4	During the past three months, how often has your use of the substances led to health, social, legal, or financial problems?	“Never”, “once or twice”, “monthly”, “weekly” or “daily or almost daily”
5	During the past three months, how often have you failed to do what was normally expected of you because of the use of a substance?	“Never”, “once or twice”, “monthly”, “weekly” or “daily or almost daily”
6	Has a friend or relative or anyone else ever expressed concern about your use of a substance?	“No, never”, “Yes, in the past three months” or “Yes, but not in the past three months”
7	Have you ever tried and failed to control, cut down, or stop using a substance?	“No, never”, “Yes, in the past three months” or “Yes, but not in the past three months”
8	Have you ever used any drug by injection?	“No, never”, “Yes, in the past three months” or “Yes, but not in the past three months”

In essence, the purpose of the questionnaire is to identify three key aspects of substance use: lifetime use of substances, recent use of substances (past three months), and problems associated with substance use such as dependence, intoxication, and social/legal issues. The responses to these questions are used to calculate a risk score for each substance. Based on the score, individuals are categorized into low-risk, moderate-risk, or high-risk groups.

By stratifying individuals into different risk levels, ASSIST allows for the implementation of interventions, ranging from brief advice to specialized treatment [38]. The different categories of risk and their recommended further management are described in Table 9. Early identification and intervention help potentially reduce the negative health and social consequences of substance use disorders [38,39].

**Table 9:** ASSIST score based risk categorization, interpretation, and recommended further management ASSIST: Alcohol, Smoking, and Substance Involvement Screening Test

Score	Risk category	Interpretation	Recommendation
0-3	Low	No significant problem or low levels of use	No intervention
4-26	Moderate	Potential issues	Further assessment with or without brief intervention
27 and above	High	Serious problem	Immediate intervention

NIDA Drug Use Screening Tool

The NIDA Drug Use Screening Tool consists of two components: NIDA Quick Screen and NIDA-modified ASSIST 2.0. Both these are screening instruments developed by NIDA to identify individuals at risk for substance use disorders, including illicit drugs and prescription medications. This tool is particularly useful in clinical settings for early identification and intervention [40,41].

NIDA Quick Screen is a brief screening tool that assesses the frequency of use of various substances, including alcohol, tobacco products, prescription drugs for non-medical reasons, and illegal drugs. Here, only one question is asked: “In the past year, how often have you used the following?” Response for each substance is recorded as “never”, “once or twice”, “monthly”, “weekly” or “daily or almost daily” [40].

NIDA-modified ASSIST 2.0 typically consists of a series of questions that assess the frequency and quantity of drug use, as well as related problems. The tool is structured along the lines of the ASSIST tool from WHO with a few minor alterations. The majority of questions address the following broad aspects: types of substances used (e.g., marijuana, cocaine, opioids), frequency of use, and consequences of use (e.g., legal issues, health problems). Responses are scored to determine the level of risk associated with substance use. Higher scores indicate greater risk and may suggest the need for further assessment or intervention, along the same lines as the ASSIST tool from WHO [41].

CAGE-AID Questionnaire

The CAGE-AID questionnaire is a widely used screening tool designed to identify individuals at risk of both alcohol and drug use disorders. It is an adaptation of the original CAGE questionnaire, which focuses solely on alcohol use; the CAGE-AID includes drug use in its spectrum as well. Similar to CAGE, CAGE-AID consists of four questions that assess both alcohol and drug use. The detailed CAGE-AID questionnaire is presented in Table 10.

**Table 10:** Details of the four-item CAGE-AID questionnaire CAGE-AID: Cut-down, Annoyed, Guilty, and Eye-opener-Adapted to Include Drugs

Acronym letter	Summarized epithet	Question format
C	Cut-down	Have you ever felt you should cut down on your drinking or drug use?
A	Annoyed	Have people annoyed you by criticizing your drinking or drug use?
G	Guilty	Have you ever felt guilty about drinking or drug use?
E	Eye-opener	Have you ever had a drink or used drugs first thing in the morning to steady your nerves or get rid of a hangover?

Each "yes" response is scored as one point, leading to a total score ranging from zero to four. A score of two or more generally suggests the need for further assessment [42,43]. The CAGE-AID questionnaire is primarily used in primary care clinical settings to quickly screen for potential substance use issues and determine whether further evaluation or intervention is necessary. The questionnaire can be administered through self-report or clinician-led interviews, allowing for some flexibility depending on the clinical milieu. The CAGE-AID questionnaire serves as an effective screening tool for alcohol and drug use disorders because of its concise format, straightforward scoring, and established psychometric properties [44].

DAST

DAST is a widely used screening tool designed to identify individuals who may have drug abuse problems. It helps healthcare providers assess the severity of drug use and its impact on daily functioning.

DAST consists of 28 "yes/no" questions that inquire about various aspects of drug use, including frequency of use, consequences of drug use (e.g., legal issues, health problems), and behavioral changes related to drug use. Each "yes" response is scored as one point, resulting in a total score of up to 28. Scores are stratified based on the risk of drug-related issues. Score and risk stratification are presented in Table 11.

**Table 11:** Risk stratification based on the results of 28-item DAST DAST: Drug Abuse Screening Test

Score	Risk category	Recommendation
0-5	Low	No intervention
6-10	Moderate	No intervention
11 and above	High	Intervention

Like all other scoring systems, DAST provides a platform for further assessment and treatment planning in addiction treatment programs and even primary healthcare settings. DAST can be administered through self-report or clinician-led interviews, allowing flexibility for use in varied healthcare settings [6,45].

Impact of COVID-19 pandemic

The COVID-19 pandemic, among other things, had a substantial negative impact on SUDs among older adults, which are best described as multifaceted. The pandemic has exacerbated existing vulnerabilities in this population, leading to increased substance use and associated health risks [2].

Why the Increase in SUDs Following COVID-19?

The COVID-19 pandemic resulted in heightened social isolation for many older adults, contributing to increased feelings of loneliness and depression. These mental health challenges often led to greater reliance on substances as coping mechanisms. Additionally, the pandemic disrupted access to healthcare services, including substance use treatment programs. Many older adults faced barriers to receiving care due to lockdowns, fear of exposure to the virus, and the transition to telehealth services, which may not have been as effective for all patients.

There has also been a noted shift in the types of substances used among older adults during the pandemic. While alcohol remains prevalent, an increase in the use of illicit drugs, including opioids and cannabis, has been reported. This shift reflects broader trends observed in younger populations but poses unique risks for older adults who may have comorbidities that complicate and are complicated by substance use [46].

Challenges in Screening and Diagnosis During the Pandemic

The pandemic highlighted significant challenges in the screening and diagnosis of SUDs among older adults. Many healthcare providers were not sufficiently well equipped to routinely screen for substance use disorders, leading to underdiagnosis. Only a small percentage of older adults entering treatment were referred by healthcare providers, indicating a gap in proactive care. Older adults are also less likely to disclose substance use due to stigma, particularly during a time when health concerns are heightened. This can lead to missed opportunities for early intervention and support [2,47].

The pandemic also underscored existing racial and socioeconomic disparities in health outcomes related to SUDs. Older adults from marginalized communities faced compounded challenges during COVID-19, including higher rates of infection and mortality, which can exacerbate stress and lead to increased substance use as a coping strategy. Socioeconomic factors also influenced access to both healthcare and substance use treatment resources, further widening the gap in care for vulnerable populations [2,48].

In summary, COVID-19 has had a profound disrupting impact on the identification and management of SUDs among older adults, highlighting the need for improved screening practices, increased awareness among healthcare providers, and targeted interventions that address the unique challenges faced by this demographic during the pandemic [2].

Treatment of SUDs in older adults

Current research indicates that the treatment approaches for SUDs in older adults can be similar to those used for younger populations. However, age-sensitive adaptations are crucial due to physiological changes associated with aging. These adaptations ensure that treatment is safe and effective for older individuals, although no randomized control trials or specific guidelines exist for this purpose [2,4].

Inpatient detoxification is often recommended for older adults, particularly those with underlying medical or mental health conditions or those who require supervised withdrawal. This approach helps manage the complexities of SUDs in this population as older adults may have heightened sensitivity to withdrawal symptoms and complications due to age-related health issues [4]. Management can be non-pharmacological or pharmacological.

Non-Pharmacologic Treatment

Non-pharmacological treatment includes motivational interviewing and psychotherapy options.

Motivational interviewing can be an effective strategy in primary care and specialty addiction clinics. It encourages patients to explore triggers for their addiction and fosters behavior change according to their personal goals.

Three main psychotherapy options are commonly employed for substance use: cognitive behavioral therapy (CBT), group therapy, and self-help groups. CBT is recognized as the gold standard for treating stimulant use disorders in older adults, especially since there are no Food and Drug Administration (FDA)-approved pharmacological treatments specifically for this demographic. Group therapy serves to provide additional social support and share experiences among peers. Self-help groups like Alcoholics Anonymous (AA) and Narcotics Anonymous (NA) can be very beneficial during extended recovery [2,4].

Pharmacologic Treatment

While pharmacologic treatments have been extensively studied in younger adults, research specific to older adults is limited. For alcohol use disorders, medications such as disulfiram, acamprosate, and naltrexone are indicated in the elderly. Options for nicotine dependence include bupropion, varenicline, and nicotine replacement therapies (gums and transdermal patches). Buprenorphine, naloxone, and methadone may be used for treating opioid, methamphetamine, and heroin use disorders. Commonly, for benzodiazepine dependence, careful medical supervision with a slow tapering schedule for at least four weeks is recommended to minimize withdrawal risks [4].

Challenges in Pharmacologic Treatment

There are currently no randomized controlled trials specifically studying pharmacologic treatments of SUDs in older adults. This lack of data underscores the need for more targeted research to establish safe and effective treatment protocols tailored to this age group [4].

The treatment of SUDs in older adults necessitates a comprehensive approach that considers the unique physiological, psychological, and social factors affecting this population. In-patient treatment may be preferred in certain cases, while non-pharmacologic strategies like motivational interviewing and CBT play crucial roles in recovery. As the prevalence of SUDs among older adults continues to rise, it is essential to adapt treatment modalities to meet their specific needs effectively [2].

Precautions essential in older adults while using the common medications for SUDs are given in Table 12 (adapted from [4]).

**Table 12:** Commonly used pharmacological agents and their precautions in the elderly CAD: Coronary artery disease; MAO: Monoamine oxidase; CKD: Chronic kidney disease; CLD: Chronic liver disease; NRT: Nicotine replacement therapy

Substance of abuse	Pharmacologic agent	Precautions
Alcohol	Disulfiram	Increased fall risk
	Acamprosate	Avoid in CKD
	Naltrexone	Avoid in CLD. Avoid with concomitant opioid therapy. Increased fall risk. Decreases appetite
Nicotine	Bupropion	Avoid in seizures, psychosis, and eating disorders. Avoid in concomitant MAO inhibitor therapy. Lower dose and frequency in the elderly, particularly in CKD and CLD
	Varenicline	Adjust dosage and frequency in CKD. May increase the risk of heart attack in CAD. Limited data on general safety in the elderly
	NRT	Safest agent in older adults
Opiate and other narcotics	Buprenorphine	Avoid in concomitant opioid analgesics
	Naloxone	Avoid in concomitant opioid analgesics
	Methadone	Avoid in concomitant opioid analgesics. Variable pharmacokinetics - difficult to titrate and prolonged half-life. Prolongation of QT interval. Risk of drug interactions

## Conclusions

The rise in unhealthy substance use among older adults is a significant public health concern, contributing to increased overdose mortality, healthcare costs, and individual and community suffering. With more older adults engaging in substance use, prevention and screening have become critical yet challenging tasks. Physiological and social changes associated with aging require tailored screening approaches to effectively identify and address substance use in this demographic. It is also essential for healthcare providers to use sensitive and appropriate language when discussing substance use and SUDs with older patients.

Moreover, the compounded effects of substance use in the context of chronic medical conditions, geriatric health issues, and extensive medication use need further exploration to fully understand the risks and impacts on older adults. Prioritizing research in this area is essential to better inform healthcare strategies and improve outcomes for this vulnerable population.
